# Methadone Access for Opioid Use Disorder During the COVID-19 Pandemic Within the United States and Canada

**DOI:** 10.1001/jamanetworkopen.2021.18223

**Published:** 2021-07-23

**Authors:** Paul J. Joudrey, Zoe M. Adams, Paxton Bach, Sarah Van Buren, Jessica A. Chaiton, Lucy Ehrenfeld, Mary Elizabeth Guerra, Brynna Gleeson, Simeon D. Kimmel, Ashley Medley, Wassim Mekideche, Maxime Paquet, Minhee Sung, Melinda Wang, R. O. Olivier You Kheang, Jingxian Zhang, Emily A. Wang, E. Jennifer Edelman

**Affiliations:** 1 Department of Internal Medicine, Yale School of Medicine, New Haven, Connecticut; 2 British Columbia Center on Substance Use, Department of Medicine, University of British Columbia, Vancouver, British Columbia, Canada; 3 Yale School of Nursing, Orange, Connecticut; 4 Vassar College, Poughkeepsie, New York; 5 Sections of General Internal Medicine and Infectious Diseases, Department of Medicine, Boston University School of Medicine and Boston Medical Center, Boston, Massachusetts; 6 Faculty of Pharmacy, Université de Montréal, Montréal, Canada; 7 VA Connecticut Healthcare System, West Haven; 8 Center for Interdisciplinary Research on AIDS, Yale School of Public Health, New Haven, Connecticut

## Abstract

**Importance:**

Methadone access may be uniquely vulnerable to disruption during COVID-19, and even short delays in access are associated with decreased medication initiation and increased illicit opioid use and overdose death. Relative to Canada, US methadone provision is more restricted and limited to specialized opioid treatment programs.

**Objective:**

To compare timely access to methadone initiation in the US and Canada during COVID-19.

**Design, Setting, and Participants:**

This cross-sectional study was conducted from May to June 2020. Participating clinics provided methadone for opioid use disorder in 14 US states and territories and 3 Canadian provinces with the highest opioid overdose death rates. Statistical analysis was performed from July 2020 to January 2021.

**Exposures:**

Nation and type of health insurance (US Medicaid and US self-pay vs Canadian provincial).

**Main Outcomes and Measures:**

Proportion of clinics accepting new patients and days to first appointment.

**Results:**

Among 268 of 298 US clinics contacted as a patient with Medicaid (90%), 271 of 301 US clinics contacted as a self-pay patient (90%), and 237 of 288 Canadian clinics contacted as a patient with provincial insurance (82%), new patients were accepted for methadone at 231 clinics (86%) during US Medicaid contacts, 230 clinics (85%) during US self-pay contacts, and at 210 clinics (89%) during Canadian contacts. Among clinics not accepting new patients, at least 44% of 27 clinics reported that the COVID-19 pandemic was the reason. The mean wait for first appointment was greater among US Medicaid contacts (3.5 days [95% CI, 2.9-4.2 days]) and US self-pay contacts (4.1 days [95% CI, 3.4-4.8 days]) than Canadian contacts (1.9 days [95% CI, 1.7-2.1 days]) (*P* < .001). Open-access model (walk-in hours for new patients without an appointment) utilization was reported by 57 Medicaid (30%), 57 self-pay (30%), and 115 Canadian (59%) contacts offering an appointment.

**Conclusions and Relevance:**

In this cross-sectional study of 2 nations, more than 1 in 10 methadone clinics were not accepting new patients. Canadian clinics offered more timely methadone access than US opioid treatment programs. These results suggest that the methadone access shortage was exacerbated by COVID-19 and that changes to the US opioid treatment program model are needed to improve the timeliness of access. Increased open-access model adoption may increase timely access.

## Key Points

**Question:**

How does timely methadone access for opioid use disorder compare between the US and Canada during COVID-19?

**Findings:**

In this cross-sectional study of methadone clinics during COVID-19 in 13 US states and the District of Columbia and 3 Canadian provinces with the highest rates of opioid overdose deaths, more than 1 in 10 clinics were not accepting patients, one-third of which reported this was due to COVID-19. Canadian clinics offered appointments faster than US clinics.

**Meaning:**

These findings suggest that methadone access may be worse than previously estimated and exacerbated by COVID-19 and that Canadian clinics may provide timelier access than US opioid treatment programs.

## Teaser

This cross-sectional study examines the proportion of clinics accepting new patients with opioid use disorder and time until first appointment in the US and Canada.

## Introduction

Opioid overdose deaths are rising in the context of the COVID-19 pandemic.^1,2,3^ Timely access to medications for opioid use disorder (OUD) is critical to preventing overdose deaths,^4^ but COVID-19 may have disrupted the treatment delivery system. Methadone access may be uniquely vulnerable to disruption given the unique regulatory burden and increased risk of COVID-19 among individuals with OUD.^5,6,7^ Even small disruptions may adversely impact OUD outcomes, as access delays as short as 1 day are associated with decreased medication initiation and delays in methadone initiation are associated with increased illicit opioid use and overdose death.^4,8,9^

Several countries changed methadone provision policies in response to COVID-19. In the US, methadone for OUD can only be administered (observed medication dosing) or dispensed (take-home medication dosing) at Substance Abuse and Mental Health Service Administration (SAMHSA)–certified opioid treatment programs (OTPs). OTPs must meet multiple federal, state, and local requirements designed to minimize diversion and mandate the frequency of administration, toxicology screening, and behavioral treatment.^10^ Beginning in March 2020, SAMHSA allowed increased take-home medication and utilization of telemedicine for established patients.^11,12,13^ Canadian professional organizations also recommended increased take-home medication in March,^14^ but methadone provision was already less restricted prior to COVID-19 facilitating integration into other health care services.^15^ Specialty and primary care clinicians may prescribe methadone in-person or through telemedicine,^16^ and both administration and dispensing may occur within pharmacies.^10,17^ The flexibility of the Canadian regulatory environment allows for greater variation in the structure of methadone clinics (ie, a clinic providing a methadone order or prescription for OUD) relative to US OTPs.

Research on COVID-19 and methadone access has been limited to date. One study found the number of new patients initiating methadone was unchanged at an OTP in Seattle, Washington, after community transmission increased.^18^ Timely methadone access prior to COVID-19 was previously compared among pregnant and nonpregnant US Medicaid patients.^19,20^ To our knowledge, no prior studies have compared timely methadone access within US OTPs and Canadian clinics despite the underlying differences in delivery systems. Therefore, we assessed the timeliness of methadone access among methadone clinics within the US and Canada during COVID-19. We hypothesized more Canadian clinics would accept new patients and would offer a timelier first appointment relative to US OTPs.

## Methods

### Study Overview and Data Sources

We conducted a cross-sectional study of methadone clinics within selected US and Canadian jurisdictions between May 11 and June 17, 2020. For US jurisdictions, we obtained OTP data on May 4, 2020, from the SAMHSA behavioral health treatment services locator, which were derived from the 2019 National Survey of Substance Abuse Treatment Services.^21^ For Canadian provinces Alberta and Ontario, we obtained methadone clinic data on May 3, 2020, from the Alberta Health Services addiction and mental health service website and the ConnexOntario addiction, mental health, and problem gambling treatment services website.^22,23^ For the Canadian province of British Columbia, we obtained methadone clinic data from the January 2020 version of the British Columbia Centre directory on Substance Use Opioid Agonist Treatment Clinics.^24^ We followed the Strengthening the Reporting of Observational Studies in Epidemiology (STROBE) reporting guideline for cross-sectional studies. The Yale institutional review board determined that this study was exempt because it did not involve human participants.

### Study Sample

We included all clinics providing methadone for OUD within the US jurisdictions of Connecticut, District of Columbia, Kentucky, Maine, Massachusetts, Maryland, Michigan, Missouri, New Hampshire, Ohio, Rhode Island, Tennessee, Vermont, and West Virginia, and the Canadian provinces of Alberta, British Columbia, and Ontario, which represented the jurisdictions with the highest 2018 opioid overdose death rates within each nation (eMethods 1 in the Supplement).^1,2^ We selected 14 US and 3 Canadian jurisdictions to create the largest feasible sample of clinics of similar size within each nation. Two states, Tennessee and Missouri, had not expanded Medicaid at the time of the study.^25^ We excluded clinics serving special populations (ie, Veterans Health Administration), clinics with a wrong phone number, clinics without methadone treatment, and clinics requesting individual identification (ie, Medicaid plan number) preventing data collection (Figure).

**Figure.**
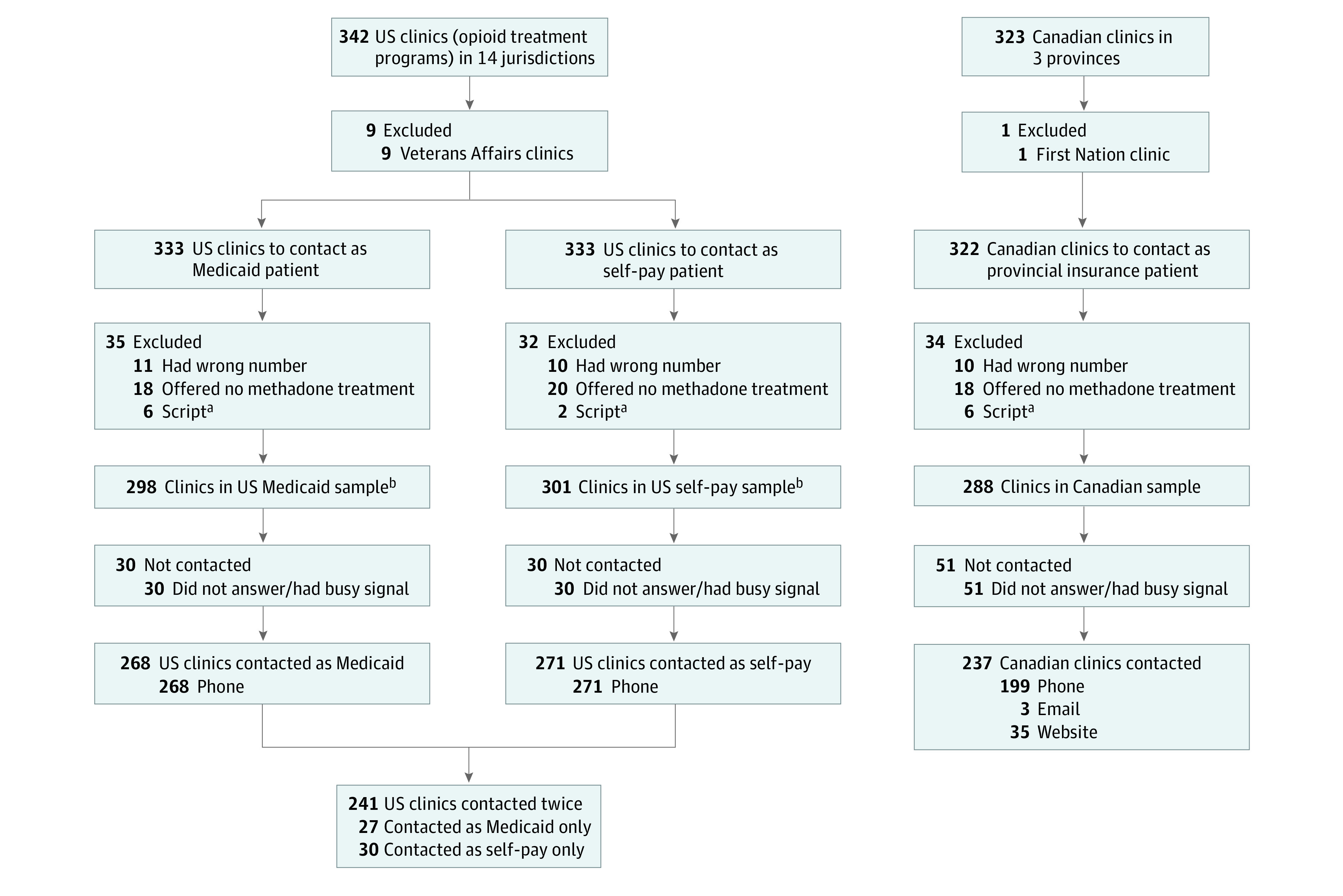
Cohort Flow Diagram of Methadone Clinics Contacted in the US and Canada in 2020 ^a^Excluded because of clinic request for individual identification (ie, Medicaid plan number) preventing data collection. ^b^Standardized patient calls were made simulating a patient aged 30 years seeking to start methadone treatment. Within the US, clinics were contacted twice: once as a patient with Medicaid and once as a patient with no health insurance (self-pay). Within Canada, clinics were contacted once as a patient with provincial insurance.

### Data Collection

Trained research team members contacted clinics at their publicly listed phone number. Consistent with prior studies,^26^ callers followed a standardized script simulating a patient aged 30 years seeking the next available appointment to initiate methadone (eMethods 2 and eMethods 3 in the Supplement). Each US clinic was contacted twice by different research team members: once as a patient with Medicaid and once as a patient with no health insurance (self-pay). In the US multiple payer system, up to two-thirds of patients pay for methadone services with Medicaid or self-pay owing to lack of health insurance.^27,28^ The Substance Use Disorder Prevention That Promotes Opioid Recovery and Treatment for Patients and Communities (SUPPORT) Act required state Medicaid programs to cover methadone treatment starting in October of 2020.^29^ Canadian clinics were contacted once as a patient with provincial insurance. Provincial insurance covers methadone-related medical visits for all citizens of a province. Clinics without successful contact (no answer or busy signal) after 3 attempts were classified as not contacted. One network of clinics in Ontario, Canada, did not answer requests by phone and its automated voice message directed callers to a website for open-access (weekly walk-in hours for new patients without appointment) information. Three clinics in this network were contacted by email to confirm the schedule. For all other clinics in this network, the next available appointment was determined according to the website.

### Outcomes

Our primary outcomes were (1) accepting new patients for methadone treatment (yes/no) and (2) wait to first appointment (days). In person and telehealth visits were accepted for the first appointment. We collected secondary outcomes to further characterize methadone provision during COVID-19. Among all clinics, secondary outcomes included (1) status of methadone treatment among clinics not accepting new patients (not accepting new patients, wait list, required inpatient detoxification, only accepts transfer from another facility), (2) not accepting new patients due to COVID-19, (3) possible to start methadone at first visit (ie, completion of clinician’s order among US OTPs or prescription among Canadian clinics), (4) open-access model, (5) clinic COVID-19 adaptations (increased take-home medication, bottle drop-off service, groups or counseling cancellation, telehealth groups or counseling, telemedicine prescribing, other), and (6) available transportation assistance. To reduce question burden, US clinics were only asked about COVID-19 adaptations during Medicaid calls.

We also collected secondary outcomes specific to nation and health insurance. Secondary outcomes for US Medicaid included (1) accepting Medicaid and (2) Medicaid first visit copay. Secondary outcomes for US self-pay included (1) accepting self-pay and (2) first visit self-pay costs. Secondary outcomes for Canadian provincial insurance included first visit copay.

Informed by implementation of an OTP open-access model,^30^ we created a secondary composite outcome to examine the proportion of clinics completing 5 cascading events necessary for timely methadone access: (1) successfully contacted clinic, (2) accepted new patients, (3) offered an appointment, (4) offered methadone at the first visit, and (5) scheduled the first visit within 1 day.

### Covariates

Clinics were classified as located in an urban or rural postal code according to the US Federal Office of Rural Health Policy and the Statistics Canada Population Centre rural area classification for postal codes.^31,32^ Because US and Canadian definitions for urban and rural were not equivalent, we did not make urban-rural comparisons.

### Statistical Analysis

First, we identified the frequency of contacted and not contacted clinics in each jurisdiction and the frequency of rural clinics by nation and insurance. We completed descriptive analyses by nation and insurance for all primary and secondary outcomes. The proportion of clinics accepting new patients among US Medicaid and US self-pay contacts were compared with Canadian contacts using a χ^2^ test. We compared the mean wait to first appointment among US Medicaid and Canadian contacts and US self-pay and Canadian contacts using a 2-sample *t* test and among US Medicaid and US self-pay contacts using a paired *t* test. Next, we estimated the proportion (cumulative probability) of clinics completing 5 cascading events for timely methadone access using a Kaplan-Meier function to account for censored observations due to non-response among secondary outcomes. We compared the proportion of clinics completing the 5 cascading events by nation and insurance using a log-rank test.

As an exploratory analysis, we compared the wait to first appointment among contacts with vs without an open-access model and among contacts reporting vs not reporting COVID-19 adaptations (telemedicine prescribing, take-home medication, and bottle drop-off service) within each nation using a 2-sample *t* test. If a COVID-19 adaptation was associated with wait to first appointment, we repeated the analysis while accounting for clinic open-access status. As a sensitivity analysis, we repeated our analyses of our primary outcomes while excluding the network of clinics not accepting phone calls. Additionally, we repeated our analysis of the proportion of clinics accepting new patients, while including all clinics publicly listed as providing methadone for OUD (including clinics with a wrong phone number and without methadone treatment) to examine the patient experience of using the service directories. We used pairwise deletion for missing primary or secondary outcome data. All hypothesis tests were 2-sided with an α of .05. We completed our analysis using Stata 15 (StataCorp) from July 2020 to January 2021.

## Results

Among 342 clinics within US jurisdictions, we excluded 9 Veterans Health Administration clinics and among 323 clinics within Canadian provinces, we excluded 1 First Nation clinic. We excluded 35 clinics from the US Medicaid sample, 32 clinics from the US self-pay sample, and 34 clinics from the Canadian sample because of a wrong phone number, lack of methadone treatment, or request for individual identification. We successfully contacted 268 clinics (90%) during US Medicaid calls, 271 clinics (90%) during US self-pay calls, and 237 clinics (82%) during Canadian calls (Table 1). Forty Medicaid contacts (15%), 43 self-pay contacts (16%), and 10 Canadian contacts (4%) were rural clinics.

**Table 1.** Geographic Characteristics of Contacted and Unable-to-Contact Methadone Clinics Within 14 US and 3 Canadian Jurisdictions in 2020

Jurisdiction and insurance type	No. (%)	
	Unable to contact^a^	Contacted
US: Medicaid (n = 298)^b^		
All US	30	268
Connecticut	2 (7)	28 (10)
District of Columbia	0	3 (1)
Kentucky	2 (7)	24 (9)
Massachusetts	2 (7)	44 (16)
Maryland	5 (17)	63 (23)
Maine	1 (3)	6 (2)
Michigan	4 (13)	31 (12)
Missouri	4 (13)	9 (3)
New Hampshire	1 (3)	5 (2)
Ohio	4 (13)	21 (8)
Rhode Island	2 (7)	13 (5)
Tennessee	1 (3)	8 (3)
Vermont	1 (3)	5 (2)
West Virginia	1 (3)	8 (3)
Rural clinic^c^	4 (13)	40 (15)
US: self-pay (n = 301)		
All US	30	271
Connecticut	3 (10)	27 (10)
District of Columbia	0	3 (1)
Kentucky	3 (10)	23 (8)
Massachusetts	5 (17)	42 (15)
Maryland	7 (23)	60 (22)
Maine	0	6 (2)
Michigan	4 (13)	33 (12)
Missouri	3 (10)	10 (4)
New Hampshire	0	7 (3)
Ohio	3 (10)	22 (8)
Rhode Island	0	14 (5)
Tennessee	0	10 (4)
Vermont	1 (3)	6 (2)
West Virginia	1 (3)	8 (3)
Rural clinic^c^	3 (10)	43 (16)
Canada: provincial insurance (n = 288)		
All Canadian	51	237
Alberta	0	24 (10)
British Columbia	22 (43)	87 (37)
Ontario	29 (57)	126 (53)
Rural clinic^c^	2 (4)	10 (4)

^a^ Clinics were designated unable to contact if no contact was made after 3 attempts (no answer or busy signal).

^b^ Standardized patient calls were made simulating a patient aged 30 years seeking to start methadone treatment. Within the US, clinics were contacted twice: once as a patient with Medicaid and once as a patient with no health insurance (self-pay). Within Canada, clinics were contacted once as a patient with provincial insurance.

^c^ Clinics were classified as located in an urban or rural postal code according to the US Federal Office of Rural Health Policy and the Statistics Canada Population Centre rural area classification for postal codes.

### Clinics Accepting New Patients and Wait to First Appointment

New patients were accepted for methadone treatment at 231 clinics (86% [95% CI, 82%-90%]) during US Medicaid contacts, 230 clinics (85% [95% CI, 80%-89%]) during US self-pay contacts, and 210 clinics (89% [95% CI, 84%-92%]) during Canadian contacts (US Medicaid vs Canadian: *P* = .41; US self-pay vs Canadian: *P* = .22) (Table 2). Among clinics not accepting new patients, 20 of 37 US Medicaid contacts (54%), 20 of 42 US self-pay contacts (48%), and 12 of 27 Canadian clinics (44%) reported not accepting new patients because of COVID-19.

**Table 2.** Status of Methadone Treatment and Days to First Appointment Among Contacted Methadone Clinics Within 14 US and 3 Canadian Jurisdictions in 2020

Outcome	No. (%)			*P* value
	US		Canada, Provincial insurance	
	Medicaid^a^	Self-pay		
Contacted clinics, No.	268	271	237	NA
Status of methadone				
Accepting new patients	231 (86)	230 (85)	210 (89)	NA
Not accepting new patients^b^	20 (7)	22 (8)	25 (11)	NA
Wait list	8 (3)	9 (3)	1 (0.5)	NA
Require inpatient detoxification^c^	2 (1)	2 (1)	0 (0)	NA
Only accepts transfers^d^	7 (3)	8 (3)	1 (0.5)	NA
Contacted clinics reporting on appointment availability, No.	257	268	237	NA
Offered appointment	190 (74)	193 (72)	196 (83)	NA
Days to first appointment				
Median (IQR)	2 (1-4)	3 (1-5)	1 (1-3)	NA
Mean (95% CI)	3.5 (2.9-4.2)	4.1 (3.4-4.8)	1.9 (1.7-2.1)	<.001^e^

Abbreviation: IQR, interquartile range.

^a^ Standardized patient calls were made simulating a patient aged 30 years seeking to start methadone treatment. Within the US, clinics were contacted twice: once as a patient with Medicaid and once as a patient with no health insurance (self-pay). Within Canada, clinics were contacted once as a patient with provincial insurance.

^b^ Methadone treatment available but not currently accepting new patients for unspecified reasons.

^c^ Only accepts new patients after completion of inpatient detoxification.

^d^ Only accepts new patients transferred from another facility.

^e^ Two-sample *t* test with unequal variance for US Medicaid vs Canada and US self-pay vs Canada.

Among clinics reporting on appointment availability, 190 US Medicaid contacts (74%), 193 US self-pay contacts (72%), and 196 Canadian contacts (83%) offered to schedule an appointment. Reasons for not offering an appointment included (1) scheduler was not available or (2) administrative requirements. Among clinics offering an appointment, 157 of 178 US Medicaid contacts (88%), 150 of 179 US self-pay contacts (84%), and 137 of 146 Canadian clinics (94%) reported it was possible to start methadone at the first appointment.

The mean wait to first appointment was greater among US Medicaid contacts (3.5 days [95% CI, 2.9-4.2 days]) and US self-pay contacts (4.1 days [95% CI, 3.4-4.8 days]) than among Canadian contacts (1.9 days [95% CI, 1.7-2.1 days]) (*P* < .001) (US Medicaid vs US self-pay: *P* = .32). The proportion of clinics providing timely access (successfully contacted clinic, accepted new patients, offered an appointment, offered methadone at the first visit, and scheduled the first visit within 1 day) was lower among US Medicaid contacts (24% [95% CI, 19%-30%]) and US self-pay contacts (20% [95% CI, 16%-25%]) than among Canadian clinics (46% [95% CI, 39%-52%]; *P* < .001) (eTable 1 and eFigure in the Supplement).

### Open-Access Model and COVID-19 Adaptations

Open-access model utilization was reported by 57 Medicaid contacts (30%), 57 self-pay contacts (30%), and 115 Canadian contacts (59%) offering an appointment. The open-access model was associated with a shorter wait to first appointment relative to clinics without an open-access model in both nations (Table 3). To adapt services to COVID-19, more than 50% of US Medicaid and Canadian contacts reported providing telemedicine prescribing (Table 4). Adaptations such as increased take-home medication allowance, bottle drop-off services, and telehealth for groups and counseling were reported more frequently among US vs Canadian contacts. Among US clinics without an open-access model, adoption (vs no adoption) of increased take-home medication was associated with a shorter wait to first appointment (Table 3). More than half (57%; 100 contacts) of US Medicaid contacts, 86% (143 contacts) of US self-pay contacts, and 84% (104 contacts) of Canadian contacts provided no transportation assistance (eTable 2 in the Supplement).

**Table 3.** Days to First Appointment for Methadone by Clinic Open Access Model or COVID-19 Adaptation Among US and Canadian Methadone Clinics in 2020

Variable	Days to first appointment				
	No.	Yes, mean (95% CI)	No.	Yes, mean (95% CI)	*P* value
Open access model^a^					
US Medicaid^b^	57	1.9 (1.5-2.4)	133	4.2 (3.3-5.1)	<.001
US self-pay	57	2.5 (1.9-3.0)	136	4.8 (3.8-5.7)	<.001
Canadian	115	1.6 (1.3-1.8)	80	2.3 (1.9-2.8)	.003
Telemedicine prescribing^c^					
US Medicaid	73	3.4 (2.5-4.4)	77	3.3 (2.5-4.2)	.89
Canadian	59	2.1 (1.6-2.6)	54	1.7 (1.3-2.1)	.23
Increased take-home medications^d^					
US Medicaid	52	2.6 (1.9-3.3)	100	3.8 (2.9-4.7)	.04
Increased take-home medications by clinic open access status					
US Medicaid with open access	14	2.0 (1.0-3.0)	31	1.7 (1.2-2.3)	.62
US Medicaid without open access	38	2.8 (1.9-3.7)	69	4.7 (3.5-5.9)	.01
Bottle drop-off service^d^					
US Medicaid	17	3.7 (1.4-6.0)	133	3.3 (2.7-4.0)	.72

^a^ Weekly walk-in hours for new patients without appointment.

^b^ Standardized patient calls were made simulating a patient aged 30 years seeking to start methadone treatment. Within the US, clinics were contacted twice: once as a patient with Medicaid and once as a patient with no health insurance (self-pay). Within Canada, clinics were contacted once as a patient with provincial insurance.

^c^ To reduce question burden, US clinics were not asked about COVID-19 adaptations during self-pay calls.

^d^ Adaptations (take-home medication and bottle drop-off service among Canadian clinics were too infrequent for comparison).

**Table 4.** Adaptations to COVID-19 Among Methadone Clinics in the US and Canada in 2020

Adaptation	No. (%)	
	US Medicaid (n = 189)^a^	Canadian provincial insurance (n = 123)
Increased take-home medication^b^	59 (31)	3 (2)
Bottle drop-off service^c^	18 (10)	1 (1)
Groups/counseling cancellation	31 (16)	2 (2)
Telehealth groups/counseling	88 (47)	6 (5)
Telemedicine prescribing	96 (51)	68 (55)
Other adaptation	126 (67)	75 (61)

^a^ Standardized patient calls were made simulating a patient aged 30 years seeking to start methadone treatment. Within the US, clinics were contacted twice: once as a patient with Medicaid and once as a patient with no health insurance (self-pay). Within Canada, clinics were contacted once as a patient with provincial insurance. To reduce question burden, US clinics were not asked about COVID-19 adaptations during self-pay calls.

^b^ Increased allowance of take-home medication.

^c^ Service to deliver methadone to the patient in the community.

### First Visit Costs

Among US Medicaid contacts, 198 of 225 (88%) accepted Medicaid and 157 of 167 (94%) required no copayment. The median (IQR) first visit Medicaid copay was US $25 (US $12-$70). The median (IQR) first visit cost among clinics not accepting Medicaid was US $75 (US $16-$140). During US self-pay contacts, 217 of 228 (95%) allowed first visit self-pay at a median (IQR) of US $87 (US $18-$144). Among Canadian contacts, 13 of 168 (8%) reported a median (IQR) first visit copay of CAD $3.75 (CAD $2-$68) (US $2.72 on May 30, 2020).

### Sensitivity Analysis

After exclusion of the 38 clinics not scheduling by phone, our primary outcomes were consistent. New patients were accepted at 173 of 199 Canadian contacts (87% [95% CI, 81%-91%]) with a mean of 2.0 days to first appointment (95% CI, 1.7-2.2 days). Upon inclusion of all clinics publicly listed as providing methadone, 231 of 285 clinics during US Medicaid contacts (81% [95% CI, 76%-85%]), 230 of 290 clinics during US self-pay contacts (79% [95% CI, 74%-83%]), and 210 of 255 clinics during Canadian contacts (82% [95% CI, 77%-87%]) accepted new patients.

## Discussion

In this cross-sectional study of methadone clinics during COVID-19 in 13 US states and the District of Columbia and 3 Canadian provinces with the highest rates of opioid overdose deaths, more than 1 in 10 clinics were not accepting new patients. More than one-third of clinics not accepting new patients reported that this was due to COVID-19. Canadian methadone clinics offered appointments faster than US OTPs. Fewer than half of Canadian clinics and fewer than one-quarter of US clinics (Medicaid or self-pay) offered appointments with the possibility of starting methadone within 1 day of contact. Together, our results suggest that a greater portion of Canadian methadone clinics provide timely access to methadone relative to US OTPs, but impediments to timely access are present at most clinics in both nations.

Although rates of methadone initiation were unchanged at an OTP serving 2630 patients in Seattle, Washington, during COVID-19,^18^ methadone clinics in both nations reported not accepting new patients due to COVID-19 despite adaptation efforts. Our results are consistent with other reports of disruptions to treatment services by individuals with substance use disorder during COVID-19.^33^ Our results suggest that previous studies overestimate methadone availability when all clinics are assumed to accept new patients.^34,35,36,37^ Importantly, people using public treatment directories to initiate methadone during the study period had approximately a 1 in 5 chance of engaging with a clinic not accepting new patients and innovations are needed to improve the quality of methadone service information.

Relative to Canadian clinics, we found the US approach of limiting methadone provision to specialized OTPs was not associated with more timely access to care. The mean difference in wait to first appointment between US OTPs and the less-regulated Canadian clinics was approximately 2 days, and previous research suggests this difference is clinically meaningful as patients with same-day access have 7.5 times the odds of arriving for their medication initiation visit relative to 2 or more days wait.^8,9^ Our results suggest explanations for the observed difference in wait times. Uptake of an open-access model was greater in Canada and was associated with more timely methadone access in both nations. Open-access model adoption does not require removal of regulatory restrictions and US and Canadian agencies should consider implementation interventions and reimbursement changes to expand adoption.^38^ Delays in access due to insufficient clinic capacity may be more likely within the US relative to Canada,^36^ which has allowed methadone provision within a greater variety of clinical settings and has reduced regulatory restrictions in response to the overdose epidemic.^15^ The greater complexity of the US multiple payer system may also delay access relative to Canada, and the cost of a first appointment may create an additional barrier within the US. Three months prior to the SUPPORT Act Medicaid requirement, we observed greater Medicaid acceptance among US OTPs relative to observed 2019 rates.^20^

Methadone clinics in both nations adapted their services to COVID-19. Telemedicine prescribing was reported by more than half of clinics within both nations. Although telemedicine was not associated with timely access, it may still reduce COVID-19 transmission. That increased take-home medication allowance, bottle drop-off services, and telehealth for groups and counseling were reported more frequently among US vs Canadian clinics suggests US OTPs required greater adaptation because of the mandated colocation of services. Within Canada, adaptions such as bottle drop-off services may have occurred at pharmacies. Among US clinics without an open-access model, increased take-home medication allowance was associated with timelier methadone access. Relaxation of take-home medication requirements may benefit patients initiating methadone at some OTPs, and future research should examine if this policy could reduce overdose, despite the potential risk of diversion, by increasing access.

That most clinics in both nations did not offer transportation assistance adds to concern over the high travel burden of methadone provision,^34,37,39^ a problem likely exacerbated by COVID-19 public transportation disruptions.^40^ Our US results suggest transportation assistance is less available to patients without health insurance. Greater adoption of mobile methadone units (methadone provisioning vehicles) could mitigate this barrier, and the US Drug Enforcement Agency recently proposed ending a moratorium on new mobile units.^41^

### Limitations

This study has several limitations. Although we contacted more than 80% of clinics for our primary outcomes, differences between respondents and nonrespondents may bias our results for secondary outcomes with lower response rates. The self-report of COVID-19 disruptions and adaptations may result in recall bias. However, US OTPs offered an appointment to 89% of Medicaid patients during a 2019 audit study compared with the 74% observed in our study.^20^ Although relatively few US patients receiving methadone utilize private insurance, we did not examine this population. Past research suggests US private insurance is also associated with access barriers.^42^ Although this study found evidence of more timely access to methadone within Canadian provinces, it underestimates true access within Canada because primary care prescribing may occur, although this is not the norm.^43,44,45^ These results examine access at the clinic level and do not assess the impact of nearby alternate clinics. However, consideration of coordination among nearby clinics is likely to further improve timely access in Canada compared with the US given the known geographic limitations of methadone availability within the US.^34^ We report the cost of the first appointment, and the cost of the medication may be an additional cost. These results may not extend to jurisdictions with lower rates of overdose deaths. Our results represent COVID-19 disruptions during late May and early June of 2020, a time when COVID-19 cases were being reported within all study jurisdictions but initial social distancing measures were being scaled back.^46,47,48^ How disruptions evolve should be a focus of further research.

## Conclusions

More than 10% of methadone clinics within jurisdictions with the highest rates of opioid overdose deaths in the US and Canada were not accepting new patients. Canadian methadone clinics were able to offer more timely methadone access relative to US OTPs. These results suggest that COVID-19 exacerbated the shortage in methadone access and changes to the US OTP model are needed to improve the timeliness of access. Timely access may be improved with increased open-access model adoption.

## Notes

Supplement. **eMethods 1.** Identification of Clinics **eMethods 2.** Data Collection Team and Procedures **eMethods 3.** Methadone Audit Study Patient Scripts **eTable 1.** Methadone Treatment Access Cascade Among Methadone Clinics Within the US and Canada in 2020 **eTable 2.** Transportation Assistance Among Methadone Clinics During Coronavirus 2019 (COVID-19) in the US and Canada in 2020 **eFigure.** Methadone Treatment Access Cascade Among Methadone Clinics Within the US and Canada in 2020
