# Supplementary material for: Methadone Access for Opioid Use Disorder During the COVID-19 Pandemic Within the United States and Canada
Source: JAMA Netw Open. 2021 Jul 23;4(7):e2118223. doi: 10.1001/jamanetworkopen.2021.18223 (PMC8303098; doi:10.1001/jamanetworkopen.2021.18223)
Supplement: Supplement. — eMethods 1. Identification of Clinics eMethods 2. Data Collection Team and Procedures eMethods 3. Methadone Audit Study Patient Scripts eTable 1. Methadone Treatment Access Cascade Among Methadone Clinics Within the US and Canada in 2020 eTable 2. Transportation Assistance Among Methadone Clinics During Coronavirus 2019 (COVID-19) in the US and Canada in 2020 eFigure. Methadone Treatment Access Cascade Among Methadone Clinics Within the US and Canada in 2020 [file jamanetwopen-e2118223-s001.pdf]

Supplemental Online Content

Joudrey PJ, Adams ZM, Bach P, et al. Methadone access for opioid use disorder during the COVID-19 pandemic within the United States and Canada. *JAMA Netw Open.* 2021;4(7):e2118223. doi:10.1001/jamanetworkopen.2021.18223

eMethods 1. Identification of Clinics

eMethods 2. Data Collection Team and Procedures

eMethods 3. Methadone Audit Study Patient Scripts

eTable 1. Methadone Treatment Access Cascade Among Methadone Clinics Within the US and Canada in 2020

eTable 2. Transportation Assistance Among Methadone Clinics During Coronavirus 2019 (COVID-19) in the US and Canada in 2020

eFigure. Methadone Treatment Access Cascade Among Methadone Clinics Within the US and Canada in 2020

This supplemental material has been provided by the authors to give readers additional information about their work.

© 2021 Joudrey PJ et al. *JAMA Network Open.*

eMethods 1

Identification of clinics

To identify methadone clinics within the province of Ontario, we did not use search terms on the ConnexOntario website. We used the advanced search option to select clinics serving opioid use disorder clients receiving "opioid replacement therapy." Within Alberta, the Alberta Health Services addiction and mental health service website was hand searched and we included all clinics listed as "opioid dependency programs providing methadone or suboxone." Within British Columbia we included all clinics identified as providing opioid agonist treatment by the British Columbia Center on Substance Use. Within the US, we included OTPs that identified as providing methadone maintenance within the SAMHSA treatment directory. Because clinics identified as providing opioid agonist treatment could provide either methadone or buprenorphine, we excluded from our primary analysis clinics reporting upon contact they in fact did not offer methadone treatment.

eMethods 2

Data collection team and procedures

Thirteen research team members completed standardized mock patient calls. A team of eight women completed the US calls and a team of three women and two men completed the Canadian calls. The patient script represented a common presentation without evidence of other chronic medical conditions and contained identical demographic and clinical characteristics, except for gender, which changed to match the caller. Clinics were called in a random order and half of the US clinics were randomly selected to receive the Medicaid contact first. The second contact (Medicaid or self-pay) occurred at least 72 hours after. If callers encountered a busy signal for more than 2 minutes, they ended the call to avoid interfering with patient care due to high call volume and/or understaffing. To pilot the patient script, callers contacted US and Canadian clinics outside of the study jurisdictions between April 20 and May 8, 2020.

© 2021 Joudrey PJ et al. *JAMA Network Open.*

eMethods 3

Methadone audit study patient scripts
5/5/20

1) United States Medicaid patient script

Dial number: [RECORD: Call answered/ busy >2 min/ Wrong number/No answer]

Opening: "Hi, I'd like to make an appointment at [clinic name] to start treatment on methadone." [RECORD: Accepting new pts/Wait list/Not accepting new patients/Does not offer methadone maintenance]

> **If wait list:** "Do you know how long I can expect to be on waitlist?" [RECORD: number of days/unknown]
>
> **If wait list OR not accepting new patients?** "Is this because of COVID19? [RECORD: Yes/No/Unsure]
>
> **If wait list OR not accepting new patients?** "If I can't start methadone, what are my options? Can I start suboxone?" [RECORD: Yes/No/Unsure]

Likely will be asked for identifying info. (See below for back story)

Next opportunity, ask:

Insurance: "I have Medicaid; do you take that?" [RECORD: Yes/No] If CT say: HuskyHealth; DC say Healthy families; MA, say: Masshealth; MD say: Medical Assistance; ME say: MaineCare; MI say: Medical Assistance or MA; MO say: MO HealthNet; RI say: RI Medical Assistance Program; TN say: TennCare; VT say: Green Mountain Care; else, say Medicaid

> **If yes:** "Ok, I am short on cash right now, will there be a co-pay or any other costs for the first visit?"
>
> **If no:** "Ok, then can I pay out of pocket to start methadone treatment? [RECORD: Yes/No]
>
> > **If yes:** "Ok, how much does the first visit cost to start methadone?" [Cost in $/unknown]
> >
> > **If provides cost:** Does that include all charges, like for labs too?" [Yes/additional costs in $/Unknown]
> >
> > **If cost unknown:** Push them – "Is there any way you can find out for me?" I am short on cash."

If more questions asked, answer then divert as quickly as possible:

Diversion Questions: "Actually before starting paperwork, do you know when your earliest opening is?"

Offered date for first appointment? [RECORD: Yes/Open access model/No]

© 2021 Joudrey PJ et al. *JAMA Network Open.*

If Yes: [RECORD: earliest date identified mm/dd/yy]

If open access model: “Ok, when is the next available time for new methadone patients?” [RECORD: Date mm/dd]

Avoid Booking: “Oh ok, I was just hoping to get in a little sooner so I think I’ll check around and give you a call back.”

Or if appointment is offered: “Ok, let me just check to see if I can get a ride for [x day] and I’ll call you back.”

Try to specify date if given range, push for a specific day record earliest: “Oh, do you know which day it would be? Because I was actually planning to be out of town around then.”

Methadone initiation: “Also, do you know if I’ll be able to get started on methadone at the first visit?” [RECORD: Yes/No].

If no: “Do you know how long after the first time I come in that I’d probably get started with the medication?” [RECORD: Number of days/unknown]

Clinic adaptations to COVID19?: "I am nervous about the COVID19 virus, will I be able to social distance while starting methadone? what options do I have?" [RECORD (check all that apply): Telehealth medical visit, Telehealth counseling or groups, groups/counseling cancellation, Increased take home dosing, bottle drop-off service, Other adaptation, Unknown]

Travel assistance: “My friend can give me a ride to the first appointment, but I will need assistance after that. Do you have any transportation assistance?” [RECORD: None/public transport fare pass/private vehicle/ ride share company/other/Unknown]

Did the clinic screen for COVID 19 risk? [RECORD all that apply: Fever, Cough, SOB, or muscle aches, exposure to positive COVID19 cases, testing for COVID19, Other]

DO NOT MAKE AN APPOINTMENT OR LET THEM HOLD A SPOT FOR YOU

Backstory:

Name: Pick name

Birthday: (Must be 30 years old)

Phone Number: “I don’t have a good number to call back right now, my phone is out of minutes and I’m just borrowing this one. I can call you back.”

Address: “Um, I’m between houses right now. Maybe I should ask before I give my friend’s address and call you back?” [redirect to appointment] “Do you know when your soonest opening would be?”

Health Insurance: “Sorry, I don’t have it on me. I can find my card and call you back.”

Medical care organization (MCO) “I can’t remember what it is called; I’d have to get it from my card. I can call you back with it.” Insisting. If insisting, prompt them to tell you which is accepted and choose a MCO to proceed: “Do you only accept some of them?” Examples. And offers examples: “The first one sounds familiar; I think it is that one.”

No Examples. If insisting without examples, provide largest MCO per state: MA: Neighborhood Health NH: Well Sense WV: Unicare OH: CareSource MD: Priority Partners DC: Ameri Health KY: WellCare of Kentucky MI: Meridian Health plan MO: Missouri care RI: United health care TN: United healthcare

Presentation:

When is the last time you used? Yesterday

What do you take? Heroin (1 to 2 grams a day, inject 3 to 4 times a day total)

Route of administration? Inject

Tried methadone before? Yes, I was on it for a year and working well but decided to taper off and relapsed about 8 months ago

How long ago did you last take methadone? Haven't been on methadone for over 8 months

Why aren't you going back to previous methadone provider? This clinic is closer to my current residence.

What dose of methadone were you previously stabilized on? 100 mg a day

Have you tried suboxone (treatment) before? "Yes, I tried suboxone 2-3 years ago but stopped because didn't like the way it made me feel. Methadone works better for me." (Is willing to consider suboxone only if methadone not available)

What is your COVID 19 status? "I have been trying to take every precaution I can. I've been washing my hands. Staying in at my friends place and observing social distancing.

Any COVID 19 exposure? "I don't know anyone who has tested positive or reported symptoms."

Any COVID 19 testing? "I have not had an fever or cold symptoms and I have not been tested."

Do you have any other health problems? "No, I don't have any health conditions and I don't take any medications."

Pregnancy (If gender female)? "I currently have an IUD, and I am not sexually active."

Secret Shopper Script Draft: MEDICAID BE SURE TO GET THE FOLLOWING BEFORE GETTING OFF THE PHONE

Can they offer you an appointment? Yes/No If no, why?

If yes, date of appointment: mm/dd/yy

Medication at first appointment? Yes/no

Time to start methadone (if no medication at first visit): date/unknown

Any clinic-specific barriers come up? Make note

© 2021 Joudrey PJ et al. *JAMA Network Open.*

2) United States no insurance patient script

Dial number: [RECORD: Call answered/ busy >2 min/ Wrong number/No answer]

Opening: "Hi, I'd like to make an appointment at [clinic name] to start treatment on methadone." [RECORD: Accepting new pts/Wait list/Not accepting new patients/Does not offer methadone maintenance]

> **If wait list:** "Do you know how long I can expect to be on waitlist?" [RECORD: number of days/unknown]
>
> **If wait list OR not accepting new patients?** "Is this because of COVID19? [RECORD: Yes/No/Unsure]
>
> **If wait list OR not accepting new patients?** "If I can't start methadone, what are my options? Can I start suboxone?" [RECORD: Yes/No/Unsure]

Likely will be asked for identifying info. (See below for back story)

Next opportunity, ask:

Insurance: "I don't have insurance, is that ok?" [RECORD: Yes/No]

> **If yes:** "How much does the first visit cost to start methadone?" [Cost in $/unknown]
>
> **If no:** End call
>
> > **If provides cost:** Does that include all charges, like for labs too?" [Yes/additional costs in $/Unknown]
> >
> > **If cost unknown:** Push them – "Is there any way you can find out for me?" I am short on cash."

If more questions asked, answer then divert as quickly as possible:

Diversion Qestions: "Actually before starting paperwork, do you know when your earliest opening is?"

Offered date for first appointment? [RECORD: Yes/Open access model/No]

> **If Yes:** [RECORD: earliest date identified mm/dd/yy]
>
> **If open access model:** "Ok, when is the next available time for new methadone patients?" [RECORD: Date mm/dd]
>
> **Avoid Booking:** "Oh ok, I was just hoping to get in a little sooner so I think I'll check around and give you a call back."
>
> **Or if appointment is offered:** "Ok, let me just check to see if I can get a ride for [x day] and I'll call you back."

© 2021 Joudrey PJ et al. *JAMA Network Open.*

Try to specify date if given range, push for a specific day record earliest: "Oh, do you know which day it would be? Because I was actually planning to be out of town around then."

Methadone initiation: "Also, do you know if I'll be able to get started on methadone at the first visit?" [RECORD: Yes/No].

If no: "Do you know how long after the first time I come in that I'd probably get started with the medication?" [RECORD: Number of days/unknown]

Travel assistance: "My friend can give me a ride to the first appointment, but I will need assistance after that. Do you have any transportation assistance?" [RECORD: None/public transport fare pass/private vehicle/ ride share company/other/Unknown]

Did the clinic screen for COVID 19 risk? [RECORD all that apply: Fever, Cough, SOB, or muscle aches, exposure to positive COVID19 cases, testing for COVID19, Other]

DO NOT MAKE AN APPOINTMENT OR LET THEM HOLD A SPOT FOR YOU

Backstory:

Name: Pick new name

Birthday: Must be 30 years old

Phone Number: "I don't have a good number to call back right now, my phone is out of minutes and I'm just borrowing this one. I can call you back."

Address: "Um, I'm between houses right now. Maybe I should ask before I give my friend's address and call you back?" [redirect to appointment] "Do you know when your soonest opening would be?"

Insurance: "I lost my insurance when I lost my job (as a teachers assistant)."

Presentation:

When is the last time you used? Yesterday

What do you take? Heroin (1 to 2 grams a day, inject 3 to 4 times a day total)

Route of administration? Inject

Tried methadone before? Yes, I was on it for a year and working well but decided to taper off and relapsed about 8 months ago

How long ago did you last take methadone? Haven't been on methadone for over 8 months

Why aren't you going back to previous methadone provider? This clinic is closer to my current residence.

What dose of methadone were you previously stabilized on? 100 mg a day

Have you tried suboxone (treatment) before? "Yes, I tried suboxone 2-3 years ago but stopped because didn't like the way it made me feel. Methadone works better for me." (Is willing to consider suboxone only if methadone not available)

What is your COVID 19 status? "I have been trying to take every precaution I can. I've been washing my hands. Staying in at my friends place and observing social distancing.

Any COVID 19 exposure? "I don't know anyone who has tested positive or reported symptoms."

Any COVID 19 testing? "I have not had an fever or cold symptoms and I have not been tested."

Do you have any other health problems? "No, I don't have any health conditions and I don't take any medications."

Pregnancy (If gender female)? "I currently have an IUD, and I am not sexually active."

Secret Shopper Script Draft: No INSURANCE BE SURE TO GET THE FOLLOWING BEFORE GETTING OFF THE PHONE

Can they offer you an appointment? Yes/No If no, why?

If yes, date of appointment: mm/dd/yy

Medication at first appointment? Yes/no

Time to start methadone (if no medication at first visit): date/unknown

3) Canada patient script

Dial number: [RECORD: Call answered/ busy >2 min/ Wrong number/No answer]

Opening: "Hi, I'd like to make an appointment at [clinic name] to start treatment on methadone." [RECORD: Accepting new pts/Wait list/Not accepting new patients/Does not offer methadone maintenance]

> **If wait list:** "Do you know how long I can expect to be on waitlist?" [RECORD: number of days/unknown]
>
> **If wait list OR not accepting new patients?** "Is this because of COVID19? [RECORD: Yes/No/Unsure]
>
> **If wait list OR not accepting new patients?** "If I can't start methadone, what are my options? Can I start suboxone?" [RECORD: Yes/No/Unsure]

Likely will be asked for identifying info. (See below for back story)

Next opportunity, ask:

Cost: Will I have to pay for anything at my first visit to start methadone? [RECORD: Yes/No]

> **If yes:** "Ok, how much does the first visit cost to start methadone?" [Cost in $/unknown]
>
> **If provides cost:** Does that include all charges, like for labs too?" [Yes/additional costs in $/Unknown]

If cost unknown: Push them – "Is there any way you can find out for me?" I am short on cash."

If more questions asked, answer then divert as quickly as possible:

Diversion Qustions: "Actually before starting paperwork, do you know when your earliest opening is?"

Offered date for first appointment? [RECORD: Yes/Open access model/No]

If Yes: [RECORD: earliest date identified mm/dd/yy]

If open access model: "Ok, when is the next available time for new methadone patients?" [RECORD: Date mm/dd]

Avoid Booking: "Oh ok, I was just hoping to get in a little sooner so I think I'll check around and give you a call back."

Or if appointment is offered: "Ok, let me just check to see if I can get a ride for [x day] and I'll call you back."

Try to specify date if given range, push for a specific day record earliest: "Oh, do you know which day it would be? Because I was actually planning to be out of town around then."

Methadone initiation: "Also, do you know if I'll be able to get started on methadone at the first visit?" [RECORD: Yes/No].

If no: "Do you know how long after the first time I come in that I'd probably get started with the medication?" [RECORD: Number of days/unknown]

Clinic adaptations to COVID19?: "I am nervous about the COVID19 virus, will I be able to social distance while starting methadone? what options do I have?" [RECORD (check all that apply): Telehealth medical visit, Telehealth counseling or groups, groups/counseling cancellation, Increased take home dosing, bottle drop-off service, Other adaptation, Unknown]

Travel assistance: "My friend can give me a ride to the first appointment, but I will need assistance after that. Do you have any transportation assistance?" [RECORD: None/public transport fare pass/private vehicle/ ride share company/other/Unknown]

Did the clinic screen for COVID 19 risk? [RECORD all that apply: Fever, Cough, SOB, or muscle aches, exposure to positive COVID19 cases, testing for COVID19, Other]

DO NOT MAKE AN APPOINTMENT OR LET THEM HOLD A SPOT FOR YOU

Backstory:

Name: Pick name

Birthday: (Must be 30 years old)

Phone Number: "I don't have a good number to call back right now, my phone is out of minutes and I'm just borrowing this one. I can call you back."

© 2021 Joudrey PJ et al. *JAMA Network Open.*

Address: "Um, I'm between houses right now. Maybe I should ask before I give my friend's address and call you back?" [redirect to appointment] "Do you know when your soonest opening would be?"

Presentation:

When is the last time you used? Yesterday

What do you take? Heroin (1 to 2 grams a day, inject 3 to 4 times a day total)

Route of administration? Inject

Tried methadone before? Yes, I was on it for a year and working well but decided to taper off and relapsed about 8 months ago

How long ago did you last take methadone? Haven't been on methadone for over 8 months

Why aren't you going back to previous methadone provider? This clinic is closer to my current residence.

What dose of methadone were you previously stabilized on? 100 mg a day

Have you tried suboxone (treatment) before? "Yes, I tried suboxone 2-3 years ago but stopped because didn't like the way it made me feel. Methadone works better for me." (Is willing to consider suboxone only if methadone not available)

What is your COVID 19 status? "I have been trying to take every precaution I can. I've been washing my hands. Staying in at my friends place and observing social distancing.

Any COVID 19 exposure? "I don't know anyone who has tested positive or reported symptoms."

Any COVID 19 testing? "I have not had an fever or cold symptoms and I have not been tested."

Do you have any other health problems? "No, I don't have any health conditions and I don't take any medications."

Pregnancy (If gender female)? "I currently have an IUD, and I am not sexually active."

Secret Shopper Script Draft: MEDICAID BE SURE TO GET THE FOLLOWING BEFORE GETTING OFF THE PHONE

Can they offer you an appointment? Yes/No If no, why?

If yes, date of appointment: mm/dd/yy

Medication at first appointment? Yes/no

Time to start methadone (if no medication at first visit): date/unknown

Any clinic-specific barriers come up? Make note

© 2021 Joudrey PJ et al. *JAMA Network Open.*

eTable 1: Methadone treatment access cascade among methadone clinics within the US and Canada in 2020

	Timely methadone access cascade[a]				
	**Contacted clinic**	**Accepted new patients**	**Offered Appointment**	**Start methadone at first visit**	**First appointment within one day**
US Medicaid[b]					
Probability of access[c], % (95% CI)	90 (86, 93)	78 (72, 82)	67 (61, 72)	60 (54, 65)	24 (19, 30)
At risk, n	298	268	231	190	157
Censored, n	0	0	11	12	0
No access, n	30	37	30	21	93
US Self-pay					
Probability of access, % (95% CI)	90 (86, 93)	76 (71, 81)	65 (59, 70)	55 (49, 60)	20 (16, 25)
At risk, n	301	271	230	193	150
Censored, n	0	0	3	13	0
No access, n	30	41	34	30	95
Canadian					
Probability of access, % (95% CI)	82 (77, 86)	73 (67, 78)	68 (62, 73)	64 (58, 69)	46 (39, 52)
At risk, n	288	237	210	196	137
Censored, n	0	0	0	47	0
No access, n	51	27	14	12	39

a Five cascading events necessary for timely methadone access
b Standardized patient calls were made simulating a 30-year-old patient seeking to start methadone treatment. Within the US, clinics were contacted twice: once as patient with Medicaid and once as a patient with no health insurance (self-pay). Within Canada, clinics were contacted once as a patient with provincial insurance.
c Kaplan-Meier function

© 2021 Joudrey PJ et al. *JAMA Network Open.*

eTable 2: Transportation assistance among methadone clinics during coronavirus 2019 (COVID-19) in the US and Canada in 2020

	**United States**		**Canada**
Transportation assistance, n (%)	**Medicaid[a] (n = 175)**	**Self-pay (n = 167)**	**Provincial insurance (n = 124)**
Public transportation fare[b]	17 (10)	5 (3)	5 (4)
Private vehicle service	7 (4)	6 (4)	1 (1)
Ride share company[c]	2 (1)	1 (1)	0 (0)
Other	53 (30)	12 (7)	14 (11)
None	100 (57)	143 (86)	104 (84)

a Standardized patient calls were made simulating a 30-year-old patient seeking to start methadone treatment. Within the US, clinics were contacted twice: once as patient with Medicaid and once as a patient with no health insurance (self-pay). Within Canada, clinics were contacted once as a patient with provincial insurance.
b Clinic covered cost of public transportation fare
c Clinic covered cost of ride share (i.e. Uber or Lyft)

© 2021 Joudrey PJ et al. *JAMA Network Open.*

eFigure 1: Methadone treatment access cascade among methadone clinics within the US and Canada in 2020

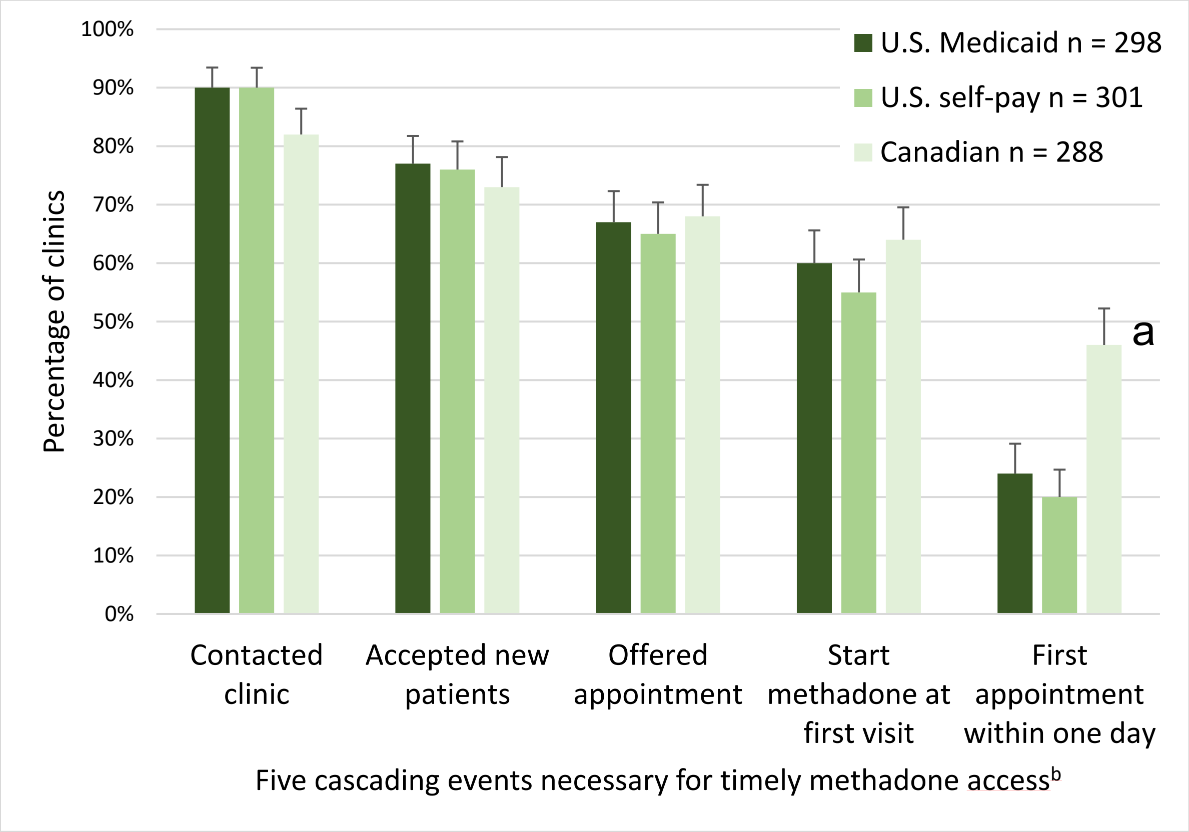

a Log rank test p <.001

b Standardized patient calls were made simulating a 30-year-old patient seeking to start methadone treatment. Within the US, clinics were contacted twice: once as patient with Medicaid and once as a patient with no health insurance (self-pay). Within Canada, clinics were contacted once as a patient with provincial insurance.

© 2021 Joudrey PJ et al. *JAMA Network Open.*
